# A Case for Two-Component Signaling Systems As Antifungal Drug Targets

**DOI:** 10.1371/journal.ppat.1004632

**Published:** 2015-02-27

**Authors:** Erika Shor, Neeraj Chauhan

**Affiliations:** 1 Public Health Research Institute, New Jersey Medical School, Rutgers, The State University of New Jersey, Newark, New Jersey, United States of America; 2 Department of Microbiology, Biochemistry and Molecular Genetics, New Jersey Medical School, Rutgers, The State University of New Jersey, Newark, New Jersey, United States of America; The University of North Carolina at Chapel Hill, UNITED STATES

## The Impact of Fungal Diseases

The recent outbreak of fungal meningitis caused by *Exserohilum rostratum* in patients receiving contaminated steroid injections resulted in 64 deaths, receiving a lot of press and briefly bringing into the public eye the difficulty of treating systemic fungal infections [1]. What is generally less well appreciated, however, is that there are several other, much more common fungal pathogens that pose a serious health threat. Indeed, currently more people die from these fungal diseases worldwide than from tuberculosis or malaria [2]. The fungal pathogens most frequently responsible for human mortality are: *Aspergillus fumigatus, Candida* spp. (predominantly *C. albicans*), *Cryptococcus neoformans, Pneumocystis carinii*, and dimorphic fungi that cause endemic mycoses (*Coccidioides immitis, Histoplasma capsulatum, Blastomyces dermatitides*, and *Paracoccidioides brasiliensis*). Fungal pathogens pose an especially high risk to individuals with compromised immunity, and this population of susceptible hosts is growing [3,4]. There has been a steady increase in the incidence of fungal infections over recent decades, primarily due to the AIDS pandemic, an increase in patients receiving cancer chemotherapy and allogeneic bone marrow transplants, a higher incidence of seriously ill patients in intensive care units, and the aging of the human population [3–8].

Despite the extensive list of fungal pathogens and the increasing frequency of their occurrence, we have at our disposal only a very limited number of antifungal drugs. The past two decades have seen the emergence of two classes of antifungals: those that target ergosterol synthesis (the azoles) and those that target cell wall β-1,3 glucan synthase (echinocandins). Of the azoles, the triazoles have gained importance as alternatives to the more toxic amphotericin B. Triazoles are fungistatic and their continued use has resulted in an increase in triazole resistance among formerly sensitive species and a rising number of disease cases caused by intrinsically azole-resistant non-*albicans Candida* species [4]. Echinocandins are fungicidal and are the drug of choice for treating most fungal infections, but these drugs are not effective in treating infections caused by *C. neoformans*, and echinocandin resistance is increasing in some *Candida* species [9]. Clearly, there is an urgent need to discover new drug targets to meet the challenges posed by fungal infections.

## Two-Component Signal Transduction Pathways in Fungal Pathogens as Potential Drug Targets

An ideal drug target is a fungal-specific protein that (1) functions as a virulence factor or is essential for fungal viability and (2) is absent from the host organism, such that its inhibition causes no toxicity in the host. The low number of drugs in our antifungal armamentarium is in part due to the relative evolutionary relatedness of fungi and mammals and the resulting paucity of fungal-specific proteins that meet these criteria. One class of molecules that do fit both of these criteria is comprised of proteins that function in the so-called two-component signal transduction pathways. These signaling pathways are based on the transfer of phosphoryl groups among their components (phosphorelays) and are one of the primary means by which bacteria and fungi sense and respond to environmental cues. Two-component signal transduction pathways present attractive targets for antifungal drug discovery because they exist in prokaryotes, plants, and lower eukaryotes but not in mammalian cells. Furthermore, while the genes encoding two-component system proteins are frequently not essential for viability, multiple studies have demonstrated the importance of two-component signal proteins for virulence in fungal pathogens, including *A. fumigatus* [10], *C. albicans* [11–13], *C. neoformans* [14], *B. dermatitidis*, and *H. capsulatum* [15] (Table 1).

**Table 1:** List of two-component signaling proteins and their functions in human fungal pathogens.

Organism	HK/HPt/RR	Gene name	Cellular role(s)	References
*A. fumigatus*	Histidine kinase	fos1	Virulence	[10]
	Histidine kinase	TcsB	Oxidative stress	[30]
	Histidine kinase	NikA/tcsC	Conidiation, hyphal growth, osmotic stress and fungicide resistance	[31]
	Transferase/HPt	Ypd1	uncharacterized	-
	Response regulator	Ssk1	Phosphorylation ofSakA MAPK	[31]
	Response regulator	Skn7	Oxidative stress	[31]
	Response regulator	Rim15	uncharacterized	-
*C. albicans*	Histidine kinase	Chk1	Quorum sensing, cell wall biogenesis, virulence, morphogenesis,stress response	[11,32–34]
	Histidine kinase	Sln1	Osmosensing, virulence	[12]
	Histidine kinase	Nik1	Morphogenesis, virulence	[12,35]
	Transferase/HPt	Ypd1	Stress response,cell membrane integrity	[36]
	Response regulator	Ssk1	Stress response, adhesion and virulence	[37–39]
	Response regulator	Skn7	Oxidative stress response, morphogenesis	[40]
	Response regulator	Srr1	Stress response, morphogenesis, apoptosis, virulence	[13,36]
	Response regulator	Rim15	uncharacterized	-
*C. neoformans*	Histidine kinase	Tco1	Negative regulator of melanin production and virulence	[14]
	Histidine kinase	Tco2	Peroxide resistance	[14]
	Histidine kinase	Tco3–7	uncharacterized	[14]
	Transferase/HPt	Ypd1	Stress response, azole drug resistance and melanin biosynthesis	[41]
	Response regulator	Ssk1	Capsule andmelanin production	[14]
	Response regulator	Skn7	Melanin production and virulence	[14,42]
*B. dermatitidis;H. capsulatum*	Histidine kinase	Drk1	Dimorphism and virulence	[15]
	Transferase/HPt	Ypd1	uncharacterized	-
	Response regulator	Ssk1	uncharacterized	-
	Response regulator	Skn7	uncharacterized	-

**Fig 1:**
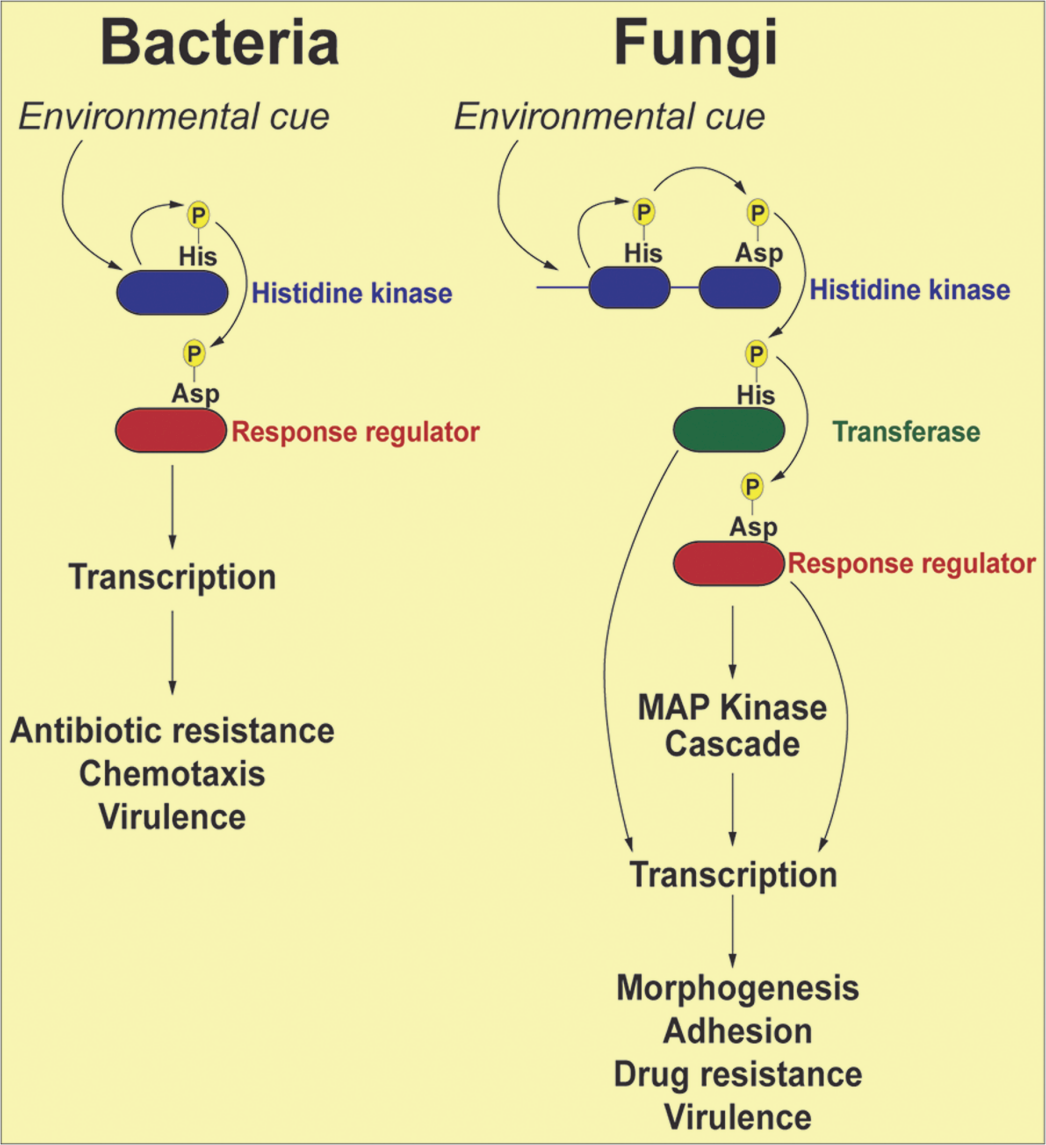
Schematic representation of two-component signal transduction pathways in bacteria and common human fungal pathogens. In response to environmental stimuli the histidine kinase (HK) is autophosphorylated on a histidine residue. In bacteria, the phosphate group from the histidine is transferred to an aspartate residue on a response regulator (RR), and the phosphorylated RR usually acts as a transcription factor to activate genes involved in response to the stimuli. In fungi, the phosphorelay involves three proteins: the phosphoryl group is first transferred intramolecularly from the histidine to an aspartate on the HK, then to a histidine on a transferase protein, and finally to an aspartate on a RR. The end result of these reactions is often activation of a downstream MAP kinase cascade, which, in turn, activates transcription factors whose target genes participate in the cellular response to environmental change.

The term “two-component” is derived from bacterial systems where the phosphorelay generally involves two proteins: a histidine kinase (HK) and a response regulator (RR) protein (Fig. 1). In response to an environmental signal, the HK, which is frequently localized in the bacterial outer membrane, is autophosphorylated on a conserved histidine residue, followed by a transfer of the phosphoryl group to a cognate response regulator protein (RR) on a conserved aspartate residue. The phosphorylated RR then usually acts directly as a transcription factor to activate genes associated with chemotaxis, stress response, quorum sensing, sporulation, virulence factor expression, and antibiotic resistance [16]. Fungal two-component phosphorelays are more intricate in two respects (Fig. 1). First, the signaling cascade involves three proteins: HK, RR, and a histidine phosphotransferase (HPt) whose function is to shuttle the phosphate moiety from HK to RR [17]. Second, in fungi, the phosphorelay typically comprises four phosphorylation events: (1) the HK is autophosphorylated on a histidine residue within its histidine kinase domain; (2) the phosphate is transferred intramolecularly to an aspartate (His→Asp) in the HK receiver domain; (3) a third, intermolecular phosphotransfer occurs to the histidine residue present in the HPt domain on the transferase (His→Asp→His); and (4) the phosphoryl group is relayed to an aspartate on the RR protein (His→Asp→His→Asp). Thus, two-component–like phosphorelay systems are unusual in terms of mechanism: the amino acids that accept phosphoryl groups are either aspartate or histidine residues. These unique features may be exploited in designing specific inhibitors that would not affect the activity of conventional Ser/Thr/Tyr kinases more prevalent in mammalian systems.

Depending on the system and the specific factors involved, the initial HK phosphorylation may occur either in response to stress or in response to removal of stress, with the ultimate outcome of either activating or down-regulating the transcription of stress response genes. A comprehensive list of all known fungal two-component signal transduction proteins and their roles in virulence-related processes can be found in Table 1. For a detailed discussion on the function of HKs and RRs in medically relevant fungi, the reader is directed to several excellent reviews [17–19].

## High Throughput Screens for Fungal Two-Component System Inhibitors

### Screening for inhibitors *in vitro*

Despite the acknowledged attractiveness of two-component systems as drug targets and their well-understood molecular mechanisms, thus far no two-component system inhibitor has reached the clinic, even though efforts to identify inhibitors of bacterial two-component systems stretch back approximately two decades. Most of the in vitro efforts to identify such inhibitors have focused on searching for compounds that could prevent HK phosphorylation activity [20]. However, many of the identified compounds did not exhibit competitive kinetics with ATP, inhibiting HK activity by other means, such as promoting HK aggregation [20]. These screens largely relied on detecting radioactively labeled phosphorylation substrates; however, phosphohistidines are highly unstable moieties [21], complicating these studies and making high-throughput screening difficult. Recently, a number of new tools have been developed to analyze HK activity, including an antibody that specifically recognizes phosphohistidine [22] and a fluorescent probe to detect histidine phosphorylation by HK in vitro [23]. The probe can label both the HK itself upon autophosphorylation and the downstream HK phosphorylation target [23], and can be thus used as a screening tool in identifying inhibitors of different steps of the phosphorelay. These tools, together with synthetic, natural, or peptide-based libraries, can facilitate rapid high-throughput screening of fungal HKs in vitro. The results of these screens can be combined with in vivo assays for two-component system activity described below.

### Screening for inhibitors *in vivo*

While in vitro screens can identify compounds that act via a desired molecular mechanism, these compounds may not be active in cellular or organismal context. For example, several identified HK inhibitors with good activity in vitro failed to inhibit bacterial growth in vivo because they were sequestered in membranes and other lipid-rich compartments [20]. Another potential benefit of in vivo screens is that they can target any component of the two-component pathway, not just the HK. To identify compounds that inhibit two-component systems in vivo, several approaches have been used in both bacteria and fungi. One set of studies focused on fungal HK Nik1 because it belongs to one of six highly conserved HK families, suggesting that its inhibition may have broad spectrum antifungal activity. In particular, one study looked for compounds that specifically inhibited the growth of a *Saccharomyces cerevisiae* strain heterologously expressing *C. albicans* Nik1 [24]. However, while this work identified two new fungicidal compounds, it also showed that these compounds did not act via inhibition of Nik1 [24].

A conceptually different type of in vivo screening approach applied in both bacteria and fungi uses a strain where the function of a particular pathway is compromised by mutation; as a result, the screen strain shows exacerbated sensitivity relative to the wild-type strain to compounds that specifically inhibit this pathway [25,26]. Because two-component system genes are not essential, in this instance, growth rate or viability may not be informative screening end points. Rather, it may be worthwhile to screen for small molecules that significantly sensitize the screen strain to oxidative stress because two-component systems are necessary for normal oxidative stress resistance in various fungi (Table 1). In a diploid fungus, such as *C. albicans*, compromising the two-component system can be achieved by deleting one of the two copies of the gene encoding a HK or RR, creating a heterozygous mutant. In a haploid fungus, such as *Candida glabrata*, a temperature-sensitive allele of a two-component system gene can be utilized for the same purpose. This approach has been used to identify compounds that affect a variety of cellular pathways, including ergosterol biosynthesis, the actin cytoskeleton, and protein folding [26], and can be readily adapted to screen for two-component system inhibitors.

Another in vivo approach can take advantage of the fact that two-component signal transduction systems regulate gene expression, either via activating downstream MAP kinase cascades or by RR proteins acting as transcription factors [19]. Thus, it should be possible to screen for pathway-specific inhibitors using a reporter whose activation or repression depends on the functionality of the two-component system pathway. Recent studies examining the effects of two-component pathways on gene expression suggest that cell-wall–maintenance genes are strongly induced by a response to stress and that this induction requires two-component systems in several fungi, including *C. albicans, S. cerevisiae*, and *C. neoformans* [27–29]. Thus, promoters of individual cell-wall–maintenance genes may be fused to fluorescent markers and used as reporters in high-throughput screens for inhibitors. This approach would likely not be specific to the two-component pathway, but will also identify inhibitors of downstream signaling events, such as other steps in the corresponding MAP kinase cascade.

## Concluding Remarks

The limited data available from studies of two-component proteins in fungal pathogens have revealed the critical functions of these proteins in adaptation to stress, regulation of virulence factors, and sensitivity to antifungal drugs, underscoring the importance of these signaling pathways in fungal pathogenesis. These features, together with the absence of two-component pathways in animals, make these proteins very attractive targets for antifungal drug discovery. Because two-component systems are found in all major fungal pathogens, drugs targeting these factors may have broad spectra. Recently developed tools for phosphohistidine analysis are likely to facilitate in vitro screening efforts, while complementary searches for pathway-based inhibitors may identify compounds that specifically inactivate two-component signal transduction in vivo.
